# 5-Aminolevulinic Acid as a Theranostic Agent for Tumor Fluorescence Imaging and Photodynamic Therapy

**DOI:** 10.3390/bioengineering10040496

**Published:** 2023-04-21

**Authors:** Richard Howley, Sharayu Chandratre, Bin Chen

**Affiliations:** 1 Department of Pharmaceutical Sciences, Philadelphia College of Pharmacy, Saint Joseph’s University, Philadelphia, PA 19104, USA; rh20580025@sju.edu (R.H.); sc20651294@sju.edu (S.C.); 2 Department of Radiation Oncology, Perelman School of Medicine, University of Pennsylvania, Philadelphia, PA 19104, USA

**Keywords:** 5-aminolevulinic acid (ALA), protoporphyrin IX (PpIX), photodynamic therapy (PDT), photodiagnosis (PD), heme biosynthesis enzymes, ferrochelatase (FECH), ATP-binding cassette subfamily G member 2 (ABCG2), fluorescence-guided tumor resection, theranostics

## Abstract

5-Aminolevulinic acid (ALA) is a naturally occurring amino acid synthesized in all nucleated mammalian cells. As a porphyrin precursor, ALA is metabolized in the heme biosynthetic pathway to produce protoporphyrin IX (PpIX), a fluorophore and photosensitizing agent. ALA administered exogenously bypasses the rate-limit step in the pathway, resulting in PpIX accumulation in tumor tissues. Such tumor-selective PpIX disposition following ALA administration has been exploited for tumor fluorescence diagnosis and photodynamic therapy (PDT) with much success. Five ALA-based drugs have now received worldwide approval and are being used for managing very common human (pre)cancerous diseases such as actinic keratosis and basal cell carcinoma or guiding the surgery of bladder cancer and high-grade gliomas, making it the most successful drug discovery and development endeavor in PDT and photodiagnosis. The potential of ALA-induced PpIX as a fluorescent theranostic agent is, however, yet to be fully fulfilled. In this review, we would like to describe the heme biosynthesis pathway in which PpIX is produced from ALA and its derivatives, summarize current clinical applications of ALA-based drugs, and discuss strategies for enhancing ALA-induced PpIX fluorescence and PDT response. Our goal is two-fold: to highlight the successes of ALA-based drugs in clinical practice, and to stimulate the multidisciplinary collaboration that has brought the current success and will continue to usher in more landmark advances.

## 1. Introduction

Light therapy has been performed for a few thousand years, dating back to the ancient civilizations of India, Greece, Egypt, and China [1]. Sunlight was used at that time as a convenient and ubiquitous tool for the treatment of diseases such as psoriasis, vitiligo, and rickets. Modern phototherapy largely stemmed from the original work of Niels Ryberg Finsen, a Danish doctor who first treated lupus vulgaris patients with sunbathing in a sun garden [2]. He later spearheaded the research for the use of UV in the treatment of tuberculosis of the skin, a feat that earned him a Nobel Prize in medicine in 1903.

Around the turn of the 20th century, Oscar Raab, a student working in the lab of Herman von Tappeiner in Munich, Germany discovered that paramecia would die after receiving acridine red dye and exposure to light. Later, Raab performed experiments that led to the discovery of the fluorescent property of molecules such as acridine. The term photodynamic action was coined from the discovery of Raab and Tappeiner. Another hallmark experiment of photodynamic action was demonstrated in 1913 by Friedrich Meyer-Betz, a German doctor who studied the phototoxic effects of hematoporphyrin, a mixture of porphyrins crudely extracted from the blood. He self-administered 200 mg of hematoporphyrin and exposed himself to the sunlight, resulting in swelling, edema, and hyperpigmentation for several months [3]. These early studies paved the way for using chemicals such as porphyrins in combination with light to address biomedical challenges.

Porphyrins such as heme in animals and chlorophyll in plants are heterocyclic macrocycle chemicals with strong absorption within the visible light spectrum [4]. They are called “the pigments of life” because they are involved in many critical biological processes such as oxygen transport, metabolism, cell signaling, and photosynthesis. All porphyrins in mammalian cells are derived from 5-aminolevulinic acid (ALA), which is synthesized in the mitochondria of almost all cells and continuously metabolized in the heme biosynthetic pathway to produce heme. The penultimate metabolite in the pathway is protoporphyrin IX (PpIX), which exhibits red fluorescence and strong photosensitizing activity upon light activation. The finding that enhanced PpIX biosynthesis can be induced by exogenously applied ALA in tumor cells has led to the use of ALA as a prodrug for PpIX-mediated photodynamic therapy (PDT) and photodiagnosis [5,6]. ALA and its derivatives have currently received worldwide approval as tumor diagnostic and therapeutic drugs (Table 1) [7]. As therapeutic agents, they are commonly used for treating superficial skin lesions including actinic keratosis and basal cell carcinoma with high clearance rates. As fluorescence imaging probes for tumor diagnosis, they are able to reveal bladder tumors and gliomas that are invisible to surgeons and facilitate tumor resection with high accuracy and precision.

The approval of ALA and its derivatives for tumor diagnosis and treatment represents a landmark in the field of photodiagnosis and PDT. It has brought tremendous success to the management of some very common pre-cancerous and cancerous conditions. However, there is still much room for further improvement to make it more effective, more precise, and more widely used. An optimized ALA protocol may deliver better outcomes by inducing higher and more selective PpIX accumulation in the target tissue. A personalized ALA protocol based on a patient’s tumor genotype and phenotype will increase the precision of this procedure. Further expanding its application to other diseases will bring benefits to more patients. In this review, we would like to summarize all U.S. FDA-approved applications of ALA and its derivatives and discuss strategies for therapeutic enhancement. Since the use of ALA-based drugs for tumor diagnosis or therapy depends on preferential PpIX tumor accumulation, we begin this review with an overview of PpIX biosynthesis from ALA and end with the prospect of combining the diagnostic and therapeutic capabilities of ALA-induced PpIX to become a fluorescent theranostic agent.

## 2. ALA-Mediated PpIX Biosynthesis in the Heme Biosynthesis Pathway

**Figure 1:**
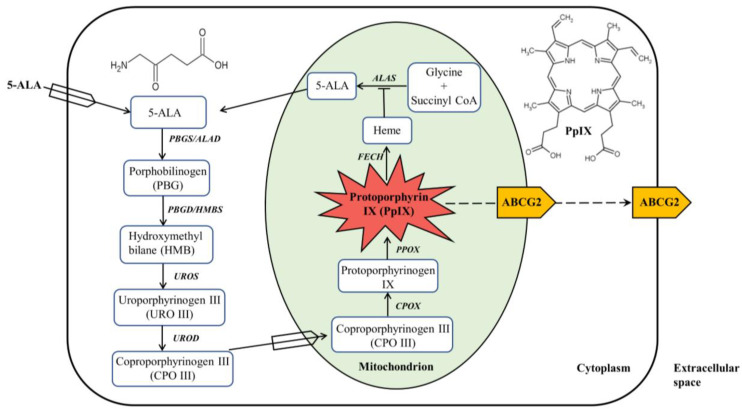
ALA-mediated PpIX biosynthesis in the heme biosynthesis pathway. ALA administered exogenously is metabolized in the heme biosynthesis pathway, which is composed of four enzymes in the cytoplasm and four enzymes in the mitochondrion, to produce PpIX with red fluorescence and photosensitizing activity. PpIX can be converted to heme with no fluorescence and photosensitizing property or effluxed out via primarily ABCG2 transporter.

Heme biosynthesis in mammalian cells is a finely tuned process that involves eight enzymes with four in the cytoplasm and four in the mitochondrion [8], as shown in Figure 1. The first step in the heme biosynthesis pathway is the synthesis of ALA from glycine and succinyl-CoA by ALA synthase (ALAS) in the mitochondria. Succinyl-CoA is a tricarboxylic acid (TCA) cycle intermediate that is derived from α-ketoglutarate (α-KG) by α-KG dehydrogenase. Glycine is the simplest amino acid that is abundant in cells for multiple biological processes. There are two tissue-specific ALAS enzymes, *ALAS1* and *ALAS2* encoded by two different genes *ALAS1* and *ALAS2*, respectively. *ALAS2* located on the X-chromosome is expressed only in the erythroid precursor cells, whereas *ALAS1* on chromosome 3 is expressed in all other types of cells. As the first intermediate in the heme biosynthesis pathway, ALA is a simple amino acid with no fluorescence and photosensitizing activity. Since the production of ALA is the rate-limiting step of heme synthesis, exogenous ALA given orally or topically bypasses this step, resulting in increased porphyrin synthesis.

The second step of heme biosynthesis is the formation of porphobilinogen (PBG) from two molecules of ALA catalyzed by ALA dehydratase (ALAD), also called porphobilinogen synthase (PBGS). This step as well as the following three enzymatic reactions all happen in the cytosol. ALAD is a tetramer of homodimers that utilizes zinc to both stabilize the protein structure and catalyze the reaction. Once four molecules of PBG are synthesized, they are linked together via deamination to form a linear tetrapyrrole named hydroxymethylbilane (HMB) by the enzyme porphobilinogen deaminase (PBGD), also called hydroxymethylbilane synthase (HMBS). HMB will be cyclized to produce the cyclic tetrapyrrole molecule uroporphyrinogen III (URO III) by uroporphyrinogen III synthase (UROS). There is a possibility that spontaneous cyclization of the HMB molecule may occur, forming uroporphyrinogen I, which will not proceed to form heme. Decarboxylation of URO III by uroporphyrinogen decarboxylase (UROD) leads to the formation of coproporphyrinogen III (CPO III), which completes the cytosolic process of heme biosynthesis. 

CPO III is then imported into the mitochondrial intermembrane space where it is converted into protoporphyrinogen IX, a reaction catalyzed by coproporphyrinogen III oxidase (CPOX). Protoporphyrinogen IX crosses the mitochondrial inner membrane and is aromatized by protoporphyrinogen oxidase (PPOX) to produce PpIX, a porphyrin metabolite with fluorescent and photosensitizing properties. Heme biosynthesis ends in the mitochondrial matrix where ferrous iron (Fe^2+^) is inserted into the tetrapyrrole core of PpIX by ferrochelatase (FECH) to form heme [8]. With the insertion of paramagnetic iron into PpIX, heme possesses neither fluorescence nor photosensitizing activity [9].

Being a ferrous iron-containing porphyrin molecule, heme is utilized as a prosthetic group in various heme-containing proteins with important biological functions [10]. In hemoglobin and myoglobin, heme functions to assist in oxygen transport and storage. In cytochromes, it functions as a catalytic group for processes such as electron transport and ATP synthesis. Additionally, heme is a catalytic necessity for enzymes involved in detoxification processes such as catalases and peroxidases [10,11]. Heme itself is the most important regulator of the heme biosynthesis pathway in that it negatively regulates heme biosynthesis via the inhibition of ALAS and stimulates heme catabolism via activating heme oxygenase 1 (HO-1) [12].

## 3. ALA as a Therapeutic Agent for PDT

PDT is a light-based therapeutic modality that involves a photosensitizer, a source of light with a specific wavelength, and molecular oxygen. These components individually are not harmful but become cytotoxic when combined due to the generation of reactive oxygen species (ROS) via type I and II photochemical reactions. Upon the absorption of light energy, photosensitizer molecules at the ground singlet state are excited to the excited singlet state. Molecules at the excited state are very short-lived and can decay back to the ground state by energy dissipation through fluorescence emission and internal conversion to heat, or undergo intersystem crossing to the excited triplet state with longer lifetime. Photosensitizer molecules at the triplet state may directly transfer electrons to substrate molecules to produce free radicals (Type I reaction) or, more commonly, transfer energy to molecular oxygen (Type II reaction), which is often abundantly available in the tissue of oxygen-consuming species, to generate single oxygen (^1^O_2_). With high reactivity, singlet oxygen can oxidize various biological substances including proteins, lipids, and nucleic acids, causing tissue oxidative damage. Due to the short lifetime and diffusion distance of singlet oxygen, the site of oxidative damage is in close proximity to the localization of the photosensitizer [13]. As a non-invasive therapeutic modality, PDT with various photosensitizers has been used in clinical settings for both cancerous and non-cancerous diseases [14]. The most commonly used photosensitizer is PpIX derived from exogenously applied ALA. 

ALA was known to cause endogenous PpIX accumulation in human lymphocytes in the 1970s [15]. In this early study, ALA-induced PpIX was much higher in lymphocytes from erythropoietic protoporphyria patients (carrying the loss-of-function mutation of the *FECH* gene) than in normal human lymphocytes and could be further enhanced by an iron chelator, suggesting the importance of *FECH* and ferrous iron in regulating PpIX level. Photoactivation of endogenous PpIX induced by ALA results in the elimination of erythroleukemia cells as Malik et al. showed in 1987, which is the first report of using ALA-PDT for cell inactivation [5]. The finding made by Kennedy et al. that ALA in aqueous solution can penetrate through abnormal keratin but not normal keratin, and induces lesion-selective PpIX in the epidermis but not in the dermis led them to conduct the first clinical trial of ALA-PDT for actinic keratosis (AK) and superficial basal cell carcinomas (BCC) [6]. In 1990, they reported an almost perfect patient response rate (complete and partial response combined) and excellent cosmetic results. Their pioneering work of applying ALA topically for stimulating local PpIX production followed by aimed light illumination for cell inactivation marked the beginning of future successes of ALA-PDT for AK and superficial BCC, the most common precancerous and cancerous skin lesions, respectively.

Multiple phase II and III clinical trials followed the initial promising study by Kennedy et al. In phase III trials of 241 patients with head AK, topical application of ALA solution on the lesion area followed by blue light treatment induced a 72% complete response rate at 12 weeks after PDT, whereas only a 20% response rate was seen in patients treated with vehicle and light only [16]. Patients were re-treated if lesions showed incomplete response or recurrence at 8 weeks after PDT. The recurrence rate between 8 and 12 weeks was 5% in all PDT-treated lesions. In contrast, 27.9% of lesions treated with vehicle and light only recurred during the same time period. With this excellent clinical performance, 20% ALA hydrochloride solution (Levulan) combined with blue light illuminator (centered at ~417 nm) received FDA approval for PDT treatment of AK in 1999, becoming the first ALA preparation entering the clinical application [17].

To increase transdermal tissue penetration for thick skin lesions, the methyl ester of ALA methyl aminolevulinate (MAL) with higher lipophilicity than ALA was evaluated for PpIX production [18]. Although MAL produces less PpIX than ALA due to the need for cleaving the ester bond to release free ALA, PpIX induced by MAL is more selective in AK lesions than that induced by ALA [19]. MAL also induces selective PpIX accumulation in thick human BCC lesions following topical application [20]. This study also evaluated the effects of MAL dose and incubation time on the intensity and depth of PpIX fluorescence and concluded that the application of 160 mg/g MAL for 3 h provided the highest ratio of PpIX fluorescence depth to tumor depth. PDT with this MAL protocol and red light illumination (with deeper tissue penetration than blue light) showed up to 91% and 97% complete responses in AK and BCC lesions, respectively, in randomized multicenter phase III clinical trials [21]. This response rate renders MAL-PDT at least equally effective as cryotherapy or excision surgery, two conventional therapies for AK and BCC. The cosmetic outcome with MAL-PDT is better than with the latter two. Based on these clinical trials, in 2004, the FDA approved PDT with 160 mg/g MAL cream (Metvixia) applied 3 h before red light (centered at ~630 nm) illumination for both thin and moderately thick AK lesions. 

Another successful approach to enhancing ALA tissue penetration is to formulate it with BF-200 nano-lipid vesicles, which increases the fusion between ALA nano-vesicles and the phospholipid bilayers of skin cells [22]. The resulting ALA nano-emulsion with 10% ALA hydrochloride (BF-200 ALA) not only leads to much stronger PpIX fluorescence than that by MAL cream but also induces fluorescence at a tissue depth of more than twice as much as that achieved with MAL cream. Multiple phase III clinical trials demonstrated that topical application of BF-200 ALA followed by red light irradiation 3 h later induced a significantly higher response rate in AK patients than PDT with 20% solution (Levulan) or 160 mg/g MAL cream (Metvixia) [23]. In 2016, BF-200 ALA gel (Ameluz) in combination with red light illumination (centered at ~635 nm) was approved by the FDA for lesion-directed and field-directed treatment of mild-to-moderate severity AK, making it the third ALA-based preparation approved for AK treatment and the only one approved for field-directed treatment.

## 4. Cell Death Mechanisms Induced by ALA-PDT

The high efficacy of ALA-PDT in clearing AK and BCC lesions, both characterized by abnormally activated cell proliferation, highlights its effectiveness in inactivating unwanted cells. ALA-PDT is very effective in inducing cell death largely due to the preferential PpIX accumulation in mitochondria, the powerhouse and stress sensor of the cell. Light activation of PpIX in mitochondria induces the formation of ROS species such as hydroxyl radicals, hydrogen peroxide, and superoxide radicals, which causes direct mitochondrial structural damage and Ca^2+^ release [24]. Consequently, the loss of mitochondrial membrane potential induces apoptotic cell death by activating the caspase-9-mediated mitochondrial apoptotic pathway, as shown in multiple studies with various cell lines [25,26,27,28]. Although produced in mitochondria, lipophilic PpIX may redistribute to other intracellular sites and, therefore, induces apoptosis via photodamage to other organelles and cellular targets. ALA-PDT is known to damage the endoplasmic reticulum (ER) and cause Ca^2+^ release, triggering apoptosis through ER-stress signaling [25]. Furthermore, blocking the activity of caspase-9 or -3 does not prevent PDT-induced apoptosis, suggesting the involvement of cell death signaling other than the mitochondria-mediated pathway. Indeed, PDT with hexaminolevulinate has been shown to induce apoptosis via both caspase-dependent and -independent pathways [29].

ALA-PDT has been shown to promote necrotic cell death as well, which is highly dependent on the cell line, PpIX localization, and PDT dose. While some cell lines undergo rapid apoptotic cell death within hours of PDT, others are more prone to die by necrosis at later times [30]. PpIX localization is an important determinant of cell death mode. PDT at a time when ALA-induced PpIX is localized in mitochondria or ER typically activates apoptotic pathways, whereas PDT at a time when PpIX is in the plasma membrane often leads to necrosis [31]. The dose of ALA and light as well as the light dose (fluence) rate may also determine how cells die of PDT, with low-dose PDT often inducing apoptosis and high-dose PDT typically resulting in necrosis [32,33].

Although necrosis in general is often considered to be a type of unprogrammed passive cell death due to extreme cell injuries, certain forms of necrosis including necroptosis, pyroptosis, and ferroptosis are highly regulated by specific pathways or are associated with damage to specific targets [34]. In LN18 human glioblastoma cells, ALA-PDT induces a form of necrosis that is highly dependent on receptor-interacting protein 3 (RIP3), a key kinase involved in necroptosis [35]. However, different from the conventional TNF-induced necroptosis where RIP3 together with RIP1, caspase-8, and FAS-associated death domain protein (FADD) form necrosomes to phosphorylate and oligomerize mixed lineage kinase domain-like pseudokinase (MLKL) for plasma membrane disruption, necrotic complexes induced by ALA-PDT do not contain caspase-8 and FADD. Additionally, there are no data showing PDT-induced MLKL phosphorylation, a hallmark of necroptosis. Thus, it remains unclear whether necroptosis, the most well-understood necrosis pathway, is involved in ALA-PDT-induced necrosis. In another study from the same group, ALA-PDT was found to induce both apoptosis and necrosis in the human osteosarcoma U2OS cell line that lacks RIP3 [36]. Surprisingly, reintroducing RIP3 significantly decreased necrosis and increased apoptosis, suggesting that RIP3 is more involved in promoting apoptotic cell death.

Lastly, ALA-PDT is also known to induce autophagy, the degradation of cellular components by lysosomes. However, instead of being a cell death mechanism, autophagy is often exploited by tumor cells to survive cancer chemotherapy and radiation therapy [37]. This is also true for ALA-PDT where autophagy has been shown to protect tumor cells from PDT-induced cell death and suppression of autophagy enhances PDT-induced cell death [38,39,40]. There is evidence suggesting that activation of tuberous sclerosis complex 2 protein is involved in the induction of pro-survival autophagy, which can be inhibited by RIP3 [41].

## 5. ALA as an Intraoperative Imaging Probe

Although ALA itself is non-fluorescent and possesses no photosensitizing capacity, PpIX produced after ALA administration exhibits red fluorescence and photosensitizing activity upon light activation. With the maximum absorption at around 405 nm in the soret band, PpIX also has multiple absorption peaks in the visible Q-bands, enabling its excitation with longer wavelengths of light to achieve deeper tissue penetration [42]. Following light excitation, PpIX emits a broad spectrum of red fluorescence emission that is centered at about 635 nm and extends beyond 700 nm. Because ALA-mediated PpIX fluorescence in tumor tissues is able to provide surgeons with a real-time guide for tumor diagnosis and margin assessment, ALA has been used as an intraoperative probe to facilitate tumor resection [43]. ALA-PpIX fluorescence-guided tumor resection has been approved for high-grade gliomas and bladder cancers where it shows better surgical outcomes than conventional white light surgery.

### 5.1. Glioma

Glioma is the most prevalent central nervous system tumor and is classified into four grades, grades I, II, III, and IV, by the World Health Organization [44]. Most gliomas are diagnosed as high-grade tumors of grades III or IV. The standard treatment for high-grade gliomas such as glioblastoma (grade IV) is surgery followed by chemotherapy and radiation therapy [45]. Despite this intensive treatment regimen, the median survival of patients with glioblastoma is only about 15 months. As the first-choice therapy, glioma surgery aims for maximum resection of tumor tissues. However, due to the invasive growth of high-grade glioma cells and the similarity in appearance between tumor and surrounding normal tissues, glioma surgery under conventional white light illumination often leaves behind viable tumor tissue, resulting in subsequent disease recurrence [46].

The first clinical trial of using ALA for PpIX fluorescence-guided glioma resection was reported by Stummer et al. in 1998 [47]. Out of 89 biopsies of grade III or IV gliomas, the sensitivity and specificity of using PpIX fluorescence for tumor detection were 85% and 100%, respectively. However, low-grade gliomas exhibited only marginal PpIX fluorescence. These promising results led Stummer et al. to conduct the first randomized phase III clinical trial and show that PpIX fluorescence-guided glioma resection resulted in significantly more gross total resection than the white light surgery (65% versus 36%) and higher 6-month progression-free survival rate (41% versus 21%) [48]. This landmark clinical trial together with additional clinical studies conducted afterward provided enough evidence for the FDA to grant the approval of ALA hydrochloride (Gleolan) in 2017 [43]. The approval allows the use of ALA as an intraoperative imaging probe to facilitate tumor resection in patients with high-grade gliomas (grade III or IV) following a standard oral administration of 20 mg/kg body weight at about 3 h prior to anesthesia. A recently completed multi-center clinical study in the U.S. further confirmed that ALA is well-tolerated and possesses high sensitivity and positive predictive value as a tumor diagnostic agent for high-grade gliomas [49]. 

Although most high-grade gliomas show strong PpIX fluorescence, low-grade gliomas typically do not exhibit PpIX fluorescence [50,51]. Nevertheless, patients with low-grade gliomas that do show PpIX fluorescence often have poor prognoses compared to patients whose tumors do not fluoresce, prompting the possible use of PpIX fluorescence as a prognostic marker for low-grade gliomas [51,52]. A comparison between fluorescing and non-fluorescing low-grade gliomas revealed significant differences in gadolinium enhancement, tumor cell proliferation, and ^18^F-fluoroethyl-l-tyrosine (^18^F-FET) PET uptake, suggesting a distinction in tumor cell biology and tumor microenvironment [52,53]. Particularly, the association between positive PpIX fluorescence and gadolinium contrast enhancement highlights the importance of the blood-brain barrier (BBB) in PpIX fluorescence positivity. ALA has a low permeability across the BBB that protects the brain from the entry of unwanted endogenous and exogenous chemicals [54]. Due to the aggressive tumor cell growth, the integrity of the BBB is often compromised in high-grade gliomas, resulting in increased ALA brain penetration and enhanced PpIX fluorescence. In contrast, ALA brain penetration is substantially limited in most low-grade gliomas where the BBB remains uncompromised, which causes reduced or even no visible PpIX fluorescence. It should be mentioned that ALA can still cross the BBB into the brain through a slow diffusional process [54]. There are studies showing that either doubling the dose of ALA to 40 mg/kg [55] or delaying the examination time to 7 and 8 h after ALA administration [53] increases the PpIX fluorescence in low-grade gliomas. 

### 5.2. Bladder Cancer

Bladder cancer is among the top 10 most common cancers in the U.S. and the 4th most common cancer in men [56]. Treatment of bladder cancer is highly dependent on the grade and stage of the disease at diagnosis. Bladder cancer at an early stage is most often treated with transurethral resection (TURBT) with the goal of removing all tumor tissues. However, bladder cancerous lesions can be flat and exhibit little difference in color and texture from the surrounding normal tissue under white light cystoscopy, making complete removal of cancerous lesions a great challenge. Residual tumor tissues after surgery will often lead to tumor recurrence and disease progression. ALA-PpIX fluorescence under blue light cystoscopy has been successfully used for the identification and removal of superficial bladder cancer. 

The first report showing that ALA is valuable for detecting bladder cancer came out in 1994 when Kriegmair et al. observed bright red fluorescence in all tumor lesions after intravesical instillation of ALA in 68 patients [57]. ALA-induced fluorescence had 100% sensitivity and 68.5% specificity for tumor detection. Notably, fluorescence detection under blue light illumination revealed 26 cancerous or precancerous lesions (out of 299 biopsies) that would have been missed by the routine white light cystoscopy. A following clinical trial demonstrated that ALA-based fluorescence cystoscopy resulted in an 18% higher diagnosis of bladder cancer than the standard white light cystoscopy, particularly dysplastic lesions and carcinoma in situ (CIS) that are superficial and flat, therefore, showing a similar appearance to the surrounding normal tissue under white light [58]. 

Since topically applied ALA has slow and limited tissue penetration due to its hydrophilicity, hexaminolevulinate (HAL) with increased lipophilicity was developed to overcome these limitations [59]. A comparison between ALA- and HAL-induced PpIX fluorescence in human bladder cancer indicates that HAL is able to induce tumor-selective fluorescence faster and at a much lower dose than ALA [60]. A phase III, multicenter clinical trial of 311 patients with superficial bladder cancer showed that fluorescence cystoscopy following bladder instillation of HAL detected at least one more tumor than white light cystoscopy in about 30% of patients [61]. A following study with 814 patients further demonstrated that transurethral tumor resection following HAL fluorescence cystoscopy significantly reduced tumor recurrence at 9 months post-surgery compared with the same procedure under white light cystoscopy [62]. Significant improvement in surgical outcomes shown in these clinical trials led to the FDA approval of HAL hydrochloride (Cysview) for the diagnosis of non-muscle (superficial) invasive bladder cancer in 2010. 

## 6. Strategies for Enhancing ALA-PpIX Fluorescence and ALA-PDT Response

**Figure 2:**
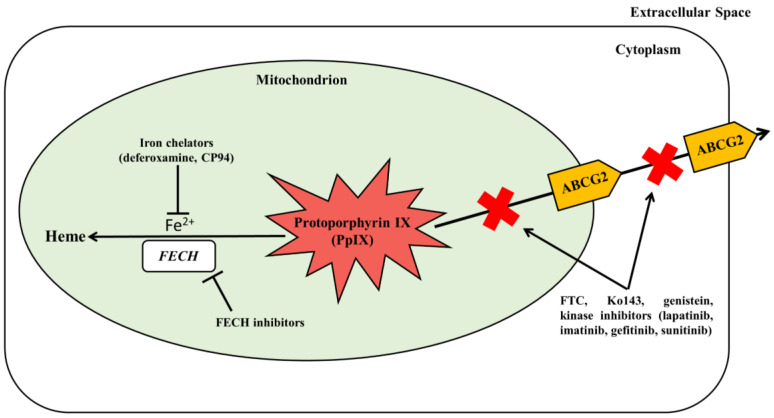
Pharmacological inhibition of PpIX-reducing biological processes for the enhancement of ALA-PpIX fluorescence and PDT. PpIX synthesized in mitochondria can be converted to heme by FECH (using Fe^2+^ as another substrate) and effluxed by ABCG2 transporter. Inhibition of these two predominant PpIX-reducing biological processes with chemical inhibitors enhances ALA-induced PpIX.

Various approaches have been explored to enhance the use of ALA and its derivatives for PpIX-mediated tumor diagnosis and treatment. Since PpIX derived from exogeneous ALA can be converted to heme (with no fluorescent and photosensitizing property) in the heme biosynthesis pathway and effluxed via active transporters, suppressing these two predominant biological processes that reduce intracellular PpIX has been extensively evaluated for increasing PpIX tumor disposition (Figure 2). The recognition that ALA-PDT activates pro-survival cell signaling pathways and antitumor immunity leads to combination treatments with inhibitors of pro-survival signaling or immune checkpoint inhibitors for therapeutic enhancement. A better understanding of tissue optics, photobiology as well as PpIX kinetics in tumor and normal tissues, has resulted in protocol refinements and device improvements for better PDT response, fewer adverse effects, and more sensitive detection of PpIX fluorescence.

### 6.1. Protocol Refinements

The FDA approval of ALA and its derivatives includes accompanying devices for PpIX activation and imaging. Blue light illuminator (centered at ~417 nm) is accompanied by 20% ALA solution (Levulan) for PDT of skin lesions in the U.S., whereas red light (centered at ~635 nm) is predominately used in Europe. PpIX has stronger absorption of blue light than of red light, while red light penetrates deeper into the tissue. ALA-PDT with blue light shows equal efficacy to PDT with red light in clearing BCC lesions at 6 months after treatment [63]. However, blue light PDT causes less pain, a major adverse effect of ALA-PDT for skin lesions. Furthermore, blue light illumination immediately after ALA application results in significantly less pain, without losing much efficacy evaluated at 3 months after treatment, than the conventional protocol where ALA is applied hours before light treatment [64]. Although the long-term response remains to be determined, this essentially painless protocol may improve patient compliance with ALA-PDT. 

PDT protocols with fractionated light illumination have been shown to enhance PDT outcomes [65]. Particularly, a double-illumination PDT scheme with 20 and 80 J/cm^2^ at 4 and 6 h after topical ALA application yields a much better clinical response than conventional single 75 J/cm^2^ illumination at 4 h after ALA. Compared with single-illumination PDT, double-illumination PDT resulted in a significantly higher complete response rate at one year after treatment in patients with AK [66] or superficial BCC [65] lesions. A follow-up study at five years after treatment still showed better responses in BCC patients treated with double-illumination PDT [67]. Although the detailed mechanism underlying increased response to double-illumination PDT remains unknown, more PpIX production and better tissue oxygenation occurring during the 2 h period between two illuminations likely increase tumor cell sensitivity to the second treatment [68,69]. 

The standard protocol of ALA for fluorescence-guided resection of high-grade glioma is oral administration at a dose of 20 mg/kg body weight about 4 h before surgery, which is based on the assumption that PpIX fluorescence will be at a maximum at this time [43]. However, real-time measurement of PpIX fluorescence in high-grade glioma patients indicates the maximum fluorescence at 7–8 h [70]. For gliomas showing weak fluorescence, the peak time of fluorescence is at 8–9 h after ALA administration. PpIX fluorescence also peaks at 7–8 h after ALA in patients with low-grade glioma [53] or meningioma [71]. These results suggest that the timing and dose of ALA need to be optimized for better tumor detection with PpIX fluorescence. This notion is supported by the finding that doubling the dose of ALA from 20 to 40 mg/kg not only significantly increases tumor PpIX fluorescence but also doubles the rate of tumor detection in low-grade glioma patients [55].

### 6.2. Device Improvements & Developments

Although blue and red light-emitting diode (LED) illuminators are commonly used as the light source to activate ALA and MAL for PDT of AK lesions, natural daylight is emerging as an attractive and convenient alternative. Multiple clinical trials have shown no significant difference in complete response rate between patients treated with daylight-PDT or conventional PDT with LED light [72]. Daylight-PDT is particularly good for patients with multiple or confluent AK lesions that are difficult to cover entirely with an LED lamp, plus it causes less adverse skin reactions and is nearly painless. To circumvent the limitation associated with natural sunlight, i.e., dependence on the time, weather, and geographic location, simulated daylight system can be used as a stable light source. In a recent clinical study, BF-200 ALA-PDT using the IndoorLux system, a simulated daylight source, was shown to be able to completely clear about 85% of lesions in AK patients with virtually no pain [73].

Improvements in current PpIX imaging devices have been made to better visualize PpIX fluorescence in gliomas. PpIX fluorescence is usually visualized with the BLUE 400 observation filter in a surgical microscope [74]. The system is composed of violet-blue light (375–440 nm) excitation and a long-pass dichroic filter to allow the transmission of the entire PpIX emission and part of blue excitation light to the observer, enabling the visualization of red PpIX fluorescence on a blue background. Designed for rapid recognition of red fluorescence emission, the system, however, provides surgeons with a dim and limited view of the background, requiring frequent switching to the white light mode for reorientation and hemostasis. To enhance background visualization, ALA is used in combination with fluorescein, a blood perfusion tracing dye with green fluorescence, for dual labeling of tumor tissues [75]. A novel filter system (YB 475) has been developed to ensure simultaneous visualization of both green fluorescence from fluorescein and red fluorescence from PpIX through the surgical microscope. With the surgical field brightened with green fluorescence, surgeons have better visualization of PpIX fluorescence as well as the background. Another approach to enhancing the background view is to modify the spectral setting of the BLUE 400 filter. Instead of using only blue light for the background illumination, the revised BLUE 400 AR filter system selects both blue and orange light to better illuminate the background [76]. Thus, the resulting images reveal more background tissue structural details, enabling surgeons to navigate and reorient during surgery.

Conventional surgical microscopes also struggle to show PpIX fluorescence in gliomas deep inside surgical cavities due to limited tissue penetration with blue light and hindered light illumination. Endoscopes with angled lenses are able to increase PpIX detection by providing focused light illumination within the cavity, resulting in the diagnosis and resection of glioblastomas that were not visible by the microscope in 10 out of 12 (83.3%) patients [77]. Incorporating this endoscope-based PpIX detection into a conventional microscope-based platform will potentially increase glioblastoma surgical outcomes by removing tumors in deep surgical cavities. 

New imaging devices are being developed and tested in clinical settings for easier and more sensitive visualization of PpIX fluorescence. As current glioma surgery is often performed with the aid of surgical loupes, a loupe device with three LED light sources has been developed for PpIX fluorescence-guided glioma resection [78]. This triple-LED loupe system includes a white LED for white light surgery, a 409-nm LED for PpIX excitation, a 450-nm LED for background illumination, magnifying lenses, and filters. Although with less magnification power, a smaller field of view, and a shorter working distance than a surgical microscope, the system offers the advantages of much brighter fluorescence, easier setup, and greater maneuverability and portability, providing a simpler and more convenient device for visualizing PpIX fluorescence. 

Additionally, current microscope-based PpIX visualization technology aims to reveal perceived differences in fluorescence intensity between tumor and normal tissues, which is sensitive to background signals and variations in tissue optical properties. To overcome the limitation of this intensity-based detection, new imaging technologies based on fluorescence lifetime [79] and tissue spectra [80] have been developed for PpIX visualization in gliomas. Other limitations associated with current PpIX imaging with wide-field surgical microscopy and blue light excitation are the lack of quantitative measurements and limited tissue penetration, respectively. Probe-based sectioning microscopes with red light illumination are able to provide high-resolution quantitative imaging of PpIX even in deep tissues [81]. The use of these more sensitive technologies may increase tumor detection in low-grade glioma, tumor borders, and surgical cavities where tumor PpIX fluorescence tends to be low. Lastly, combining PpIX fluorescence imaging, which is highly effective in visualizing tumor core, with Raman spectroscopy, which is more sensitive in detecting infiltrating tumor cells than PpIX imaging, has shown the promise of increasing tumor detection in glioblastoma patients [82].

### 6.3. Inhibition of PpIX Bioconversion

PpIX generated after ALA administration will continue to be metabolized in the heme biosynthetic pathway to produce heme with no fluorescent and photosensitizing properties. Thus, inhibition of PpIX to heme bioconversion may cause more PpIX accumulation, resulting in enhanced PpIX fluorescence and PDT response. Since this bioconversion step is catalyzed by the enzyme FECH, which uses ferrous iron as another substrate to form heme, inhibiting PpIX to heme bioconversion can be achieved by two pharmacological approaches, FECH inhibition and iron chelation (Figure 2). 

Iron chelators are commonly used to inhibit PpIX to heme bioconversion by chelating ferrous iron. Non-specific and membrane-impermeable metal ion chelator ethylenediaminetetraacetic acid (EDTA) is the first chelator shown to increase PpIX levels in cells [83] and sensitize cells to ALA-PDT [84]. However, iron-specific and membrane-permeable chelators such as deferoxamine (DFO) are more effective than EDTA in increasing PpIX accumulation [85]. DFO has been demonstrated to enhance PpIX fluorescence and/or PDT effects in various studies with different cell lines including skin, bladder, and glioblastoma tumor cells [86,87,88,89,90]. Furthermore, it significantly enhances ALA-PpIX fluorescence in glioblastoma [89] and skin cancer [69] animal models, although its effect on enhancing ALA-PDT in the skin tumor model is not statistically significant. In addition to DFO, another FDA-approved iron chelator deferasirox (DFX) has also shown strong enhancement of ALA-PpIX and PDT in both SCC-25 human squamous cell carcinoma cell line and mouse tumor model [91]. 

Although not yet FDA-approved, 1, 2-diethyl-3-hydroxypyridin-4-one (CP94) is another iron chelator that has been extensively explored for enhancing PpIX fluorescence and PDT response. It is more effective than DFO in increasing ALA- or MAL-induced PpIX accumulation in skin cancer cells likely due to its smaller size and higher lipophilicity, resulting in greater cellular uptake [92]. Its effectiveness in enhancing PpIX fluorescence and/or ALA-PDT has been demonstrated in human skin, bladder, and glioblastoma tumor cell lines [93,94,95]. ALA in combination with CP94 induces significantly higher PpIX fluorescence than ALA alone in rat bladder urothelium [96] and colonic mucosa [97]. Moreover, a small pilot clinical trial has demonstrated the safety and efficacy of combining MAL and CP94 for BCC treatment [98]. A drug conjugate called AP2-18 that links ALA to CP94 through a cleavable ester bond has been developed in order to release both agents simultaneously following the cleavage by esterases [99]. Multiple studies with different cell lines indicate that AP2-18 increases ALA-PpIX fluorescence and PDT as effectively as ALA and CP94 co-administered separately [99,100,101].

Despite these promising results from many in vitro studies, EDTA does not increase PpIX production in a mouse tumor model [102] and BCC patients [103] after ALA administration, and DFO does not enhance PpIX fluorescence in skin cancer patients either [104]. It turns out that the effects of iron chelators on ALA-PpIX fluorescence are highly dependent on the cell line and ALA dose [85,88,92,105]. While some cell lines are sensitive to PpIX enhancement by chelators, others are not. Chelator-induced PpIX enhancement is more pronounced at low doses of ALA than at the high doses of ALA that are typically used. Furthermore, our results indicate that the effects of DFO are dependent on the duration of treatment and *FECH* expression [106,107]. Particularly, DFO increases ALA-PpIX fluorescence more after 1 h than after 4 h treatment and there is an inverse correlation between PpIX fluorescence and the enhancement effect of DFO. All of these results seem to suggest that enhancement of ALA-PpIX by iron chelators is limited by the availability of ferrous iron following ALA administration. When ferrous iron is depleted, which may occur as a result of increased heme biosynthesis after ALA administration, the effect of iron chelators on PpIX enhancement is diminished.

In addition to iron chelators, inhibition of FECH activity is another approach to inhibiting PpIX to heme bioconversion. Although genetic silencing of *FECH* significantly increases ALA-PpIX fluorescence in multiple cell lines [108,109,110,111] and even in an animal study [112], pharmacological inhibition of FECH with enzymatic inhibitors for the enhancement of PpIX has not been well studied. Inhibition of FECH with NOC-18, a nitric oxide-generating reagent, enhances ALA-PpIX fluorescence and PDT response in different cell lines [113,114,115], whereas FECH inhibitor dihydropyridine 3,5-diethoxycarbonyl-1,4-dihydrocollidine reduces PpIX fluorescence [85]. Vitamin D3 (calcitriol) is believed to enhance ALA-PpIX fluorescence partially due to the downregulation of *FECH* [116]. However, in all of these studies, there is a lack of assessment of agents’ effects on FECH activity. Studies using selective and potent FECH inhibitors are needed to determine how inhibiting FECH activity affects ALA-PpIX fluorescence and PDT.

### 6.4. Inhibition of PpIX Efflux

PpIX produced after ALA administration can be effluxed out of cells, resulting in reduced intracellular PpIX levels. Therefore, inhibiting PpIX efflux may increase intracellular PpIX accumulation and sensitize cells to ALA-PDT (Figure 2). Being lipophilic, PpIX may come out of cells through exocytosis and active transporters [117]. The first evidence suggesting that PpIX is a substrate of ATP-binding cassette subfamily member G2 (ABCG2) transporter came out unexpectedly from an *Abcg2* knockout study [118]. *Abcg2*-knockout mice developed protoporphyria with increased PpIX in erythrocytes and plasma, suggesting the involvement of *Abcg2* in PpIX transport. A subsequent study determined the effects of ABCG2 overexpression and transporter inhibition on intracellular and extracellular PpIX, providing compelling evidence that PpIX is an ABCG2 substrate [119].

Identification of PpIX as a substrate of ABCG2 opens the door to using ABCG2 inhibitors to enhance ALA-PpIX fluorescence and PDT response. Fumitremorgin C (FTC) is the first selective ABCG2 inhibitor shown to significantly increase ALA-PpIX fluorescence and PDT-induced cytotoxicity in ABCG2-transfected cells [120]. It significantly increases the intracellular PpIX after ALA, while reducing the extracellular PpIX, in T24 human urothelial cancer cell line but not in its ABCG2-knockdown counterpart [114]. Its effectiveness in increasing ALA-PpIX fluorescence has been demonstrated in a variety of tumor cell lines [117]. However, the neurotoxicity caused by fumitremorgin C led to the development of its analog agent Ko143 [121], which is more widely used as a potent ABCG2 inhibitor. Ko143 is able to significantly enhance ALA-PpIX fluorescence in different human tumor cell lines including skin, bladder, breast, kidney, and brain tumors [122,123,124,125,126,127]. In addition to the increase in total PpIX fluorescence, Ko143 treatment also preferentially induces PpIX accumulation in mitochondria after ALA, therefore, sensitizing tumor cells to PDT-induced apoptosis [123,125,128]. These studies demonstrate the efficacy of ABCG2 inhibitors for enhancing ALA-PpIX fluorescence and PDT.

Ko143, unfortunately, is also not suitable for in vivo application due to its instability in the plasma and rapid inactivation in the liver [129,130]. In fact, there is no ABCG2 inhibitor currently available for clinical application despite extensive efforts [131]. Since some clinically used tyrosine kinase inhibitors are both ABCG2 substrates and inhibitors [132,133], they have been explored as potential ABCG2 inhibitors. Imatinib is the first kinase inhibitor shown to increase ALA-PpIX fluorescence and PDT in tumor cell lines through its interaction with ABCG2 [134]. Similar therapeutic enhancement can also be induced by gefitinib [135] or genistein [136] in glioma cell lines. We have screened some small molecule inhibitors in a kidney cancer cell line with robust ABCG2 activity and found six FDA-approved drugs (lapatinib, gefitinib, sunitinib, vismodegib, vemurafenib, and sorafenib) that significantly enhance intracellular ALA-PpIX fluorescence [127]. Notably, the tyrosine kinase inhibitor lapatinib not only enhances PpIX fluorescence but also increases PpIX accumulation in mitochondria, which greatly potentiates PDT-induced cell death in tumor cells that are otherwise resistant to ALA-PDT [105,128]. These results indicate that repurposing lapatinib or other existing drugs as potential ABCG2 inhibitors is a promising and practical strategy for enhancing ALA-PpIX fluorescence and PDT.

### 6.5. Targeting the Pro-Survival Signaling 

While ALA-PDT targets mitochondria and other cellular targets to induce cell death, it also activates pro-survival cell signaling pathways including nuclear factor (NF)-κB, phosphoinositide 3-kinase (PI3K), and mitogen-activated protein kinase (MAPK) signaling pathways [137]. Targeting these pro-survival signaling pathways has been shown to enhance ALA-PDT response. For instance, inhibition of the NF-κB pathway greatly sensitizes glioblastoma cells to PDT-induced necrosis [38], and targeting the PI3K pathway with inhibitor LY294002 significantly enhances PDT outcomes in esophageal tumor cells and tumor model [138]. In addition, several MAPK inhibitors increase PDT-induced cytotoxicity in a human squamous carcinoma cell line [139], as does the inhibition of nuclear factor erythroid 2-related factor 2 (NRF2) with an NRF2 inhibitor [140].

Nitric oxide (NO) produced by inducible nitric oxide synthase (iNOS) is another pro-survival signaling molecule generated after ALA-PDT [141]. PDT was shown to upregulate the level of iNOS (but not other isoforms of NOS) by several folds within hours after treatment in COH-BR1 human breast cancer cell line [142]. Reducing NO production by either a NOS inhibitor or a NO scavenger enhanced PDT-induced apoptosis, whereas increasing NO level by an exogenous NO donor prevented such enhanced apoptosis, suggesting that NO induced by ALA-PDT protects tumor cells from PDT-induced cell death. Similar findings were also shown in MDA-MB-231 breast cancer cells [143], PC-3 prostate cancer cells [144], and U-87 and U-251 glioblastoma cell lines [145]. Such increased NO production, which occurred after low doses of ALA-PDT with less than ~25% cell killing, not only prevented tumor cell death but also made surviving tumor cells more proliferative and migratory [144,145,146]. Although how PDT-induced NO promotes tumor cell survival and invasiveness remains an open question, the cause for this phenomenon was shown due to the activation of PI3K and NF-κB pathways, inhibition of which dampened the NO production and its negative biological consequences [143,147,148]. These studies highlight the potential negative outcomes of insufficient ALA-PDT and illustrate the importance of maximizing PDT-induced tumor cell killing to prevent it from happening.

### 6.6. Synergy with Immune Checkpoint Inhibitors

ALA-PDT not only directly kills tumor cells but also elicits potent immune responses with important implications in the long-term therapeutic outcome. It induces the release of inflammatory cytokines such as TNFα, which promotes the infiltration of polymorphonuclear cells particularly neutrophils that can kill tumor cells directly by ROS release and indirectly by the subsequent antitumor immunity [149,150,151]. Following neutrophil infiltration, there is an influx of lymphoid cells such as CD4 and CD8 cells into the tumor tissue, leading to the generation of a specific antitumor immune response [151]. This adaptive immunity against tumor cells is associated with the immunogenic cell death (ICD) induced by ALA-PDT, which the expression of damage-associated molecular patterns (DAMPs) molecules such as calreticulin, heat shock proteins-70, and high mobility group box 1 (HMGB1) in PDT-treated tumor cells stimulates dendritic cell maturation and promotes tumor cell antigen presentation to T lymphocytes [152,153]. 

In addition to stimulating inflammation and adaptive antitumor immunity, ALA-PDT also causes immunosuppression [154,155]. Various factors including the increase in regulatory T cells, decrease in antigen-presenting Langerhans cells, and release of immunosuppressive cytokines may contribute to PDT-induced immunosuppression [151,156]. Although immunosuppression is beneficial when PDT is used to treat autoimmune disorders or chronic inflammation [157], it may reduce the effect of PDT for cancer treatment [151,158]. Unleashing immunosuppression is an effective way of enhancing ALA-PDT. For instance, squamous cancer cells express programmed death-ligand 1 (PD-L1), an immune checkpoint protein that suppresses the T cell function, inhibition of which with an immune checkpoint inhibitor results in synergistic enhancement of the antitumor effect of ALA-PDT [156].

It is worth noting that ALA-PDT is able to selectively kill activated T cells including CD4 and CD8 cells. This is because PpIX is preferentially accumulated only in activated T cells but not resting T cells without activation after ALA incubation, resulting in the selective killing of activated T cells by PDT [159,160]. However, in antigen-presenting cells such as dendritic cells and macrophages, PpIX accumulation can occur regardless of cell activation [161,162]. These findings suggest the use of ALA-PDT for autoimmune diseases, transplantation, leukemias, and lymphomas by selectively eliminating activated T cells. A recent clinical study showed that extracorporeal photopheresis by ALA-PDT was safe and well tolerated in patients with chronic graft-versus-host disease [163].

## 7. ALA-PpIX as a Fluorescent Theranostic Agent

Theranostics is a general term used to denote any therapeutic agents or modalities that combine disease diagnosis with therapy [164]. Nuclear theranostics made by conjugating radioisotopes with targeted agents (small molecules, antibodies, etc.) possessing a high affinity for cancer molecular targets have enabled simultaneous nuclear imaging for tumor detection and radiotherapy for tumor targeting. Similarly, fluorescent theranostic agents are molecules that are capable of preferentially localizing in the target tissue and, therefore, revealing target tissues through tissue-specific fluorescence emission and initiating therapeutic action following light activation. The preferential tumor disposition of PpIX after ALA administration together with its PDT-mediated cytotoxicity renders PpIX a fluorescent theranostic agent for tumor diagnosis and therapy. The use of ALA-PpIX as a fluorescent theranostic agent is well illustrated in a clinical trial where patients with recurrent glioblastoma underwent PpIX fluorescence-guided resection first followed immediately by PDT all after a single dose of ALA administration [165]. Selective ALA-induced PpIX disposition in tumors made it possible for maximum safe resection of glioblastoma as well as targeting infiltrating tumor cells and unresectable tumor tissues in eloquent regions by PDT, resulting in promising progression-free survival in a disease with dismal prognosis. 

## 8. Conclusions and Future Perspectives

**Figure 3:**
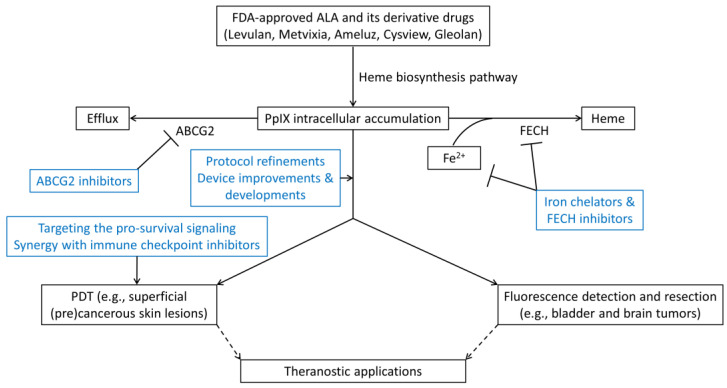
Therapeutic enhancement of ALA and its derivative drugs for PDT, fluorescence tumor detection, and resection. All ALA-based drugs are prodrugs and need to be metabolized in the heme biosynthesis pathway to produce the active drug PpIX, a fluorophore and photosensitizer. PpIX can be bioconverted to heme (with no fluorescent and photosensitizing properties) and/or effluxed out of the cell, reducing intracellular PpIX tumor accumulation. Various strategies (shown in blue) have been demonstrated to enhance the therapeutic outcomes of ALA-based drugs. Current efforts are being undertaken to combine tumor diagnostic and therapeutic capabilities of ALA-derived PpIX for theranostic applications.

Since the first approval in 1999, five ALA-based drugs have been approved by the U.S. FDA. These drugs are approved not only in the US but also in many other countries [7]. They are playing important roles in managing some common human diseases including AK (the most common precancerous lesion) and BCC (the most common cancer), and highly morbid diseases such as high-grade gliomas (Figure 3). Their applications include effective and rapid clearance of abnormally proliferative tissues as in the treatment of AK and BCC as well as facilitating the detection and removal of cancerous tissues such as bladder cancer and high-grade gliomas, all based on selective ALA-induced PpIX in the target tissue. The development of ALA-based therapeutics represents the most successful endeavor in the field of PDT and PD. Optimizing the use of these agents with strategies for enhancing ALA-PpIX fluorescence and ALA-PDT response may further improve the efficacy and therapeutic outcome.

Even though ALA-induced PpIX has the potential for being a fluorescent theranostic agent, it is currently utilized as either a PDT therapeutic agent or a fluorescent diagnostic probe. Combining its tumor diagnostic and therapeutic capabilities is appealing for the management of high-grade gliomas such as glioblastoma. As mentioned above, current PpIX visualization with wide-field surgical microscopy is highly sensitive for revealing the tumor core for bulky tumor resection, but not good for tumor visualization at the tumor margin due to low tumor cell density and surgical cavities because of the tissue hindrance to light penetration. To visualize tumor tissues in these regions, more sensitive imaging technologies such as probe-based sectioning microscopy and spectroscopy need to be deployed to aid precision surgical resection. After bulky tumor resection followed by precision tumor surgery at the tumor periphery, PDT is added to target residual infiltrating tumor cells or tumors in eloquent regions that are not safe to resect. Clinical trials are needed to determine whether such a combined procedure results in any additional benefits in patient survival compared with current standard practice.

All successful uses of ALA for tumor therapy and diagnosis depend on selective PpIX tumor disposition and there are currently substantial efforts going on to further increase tumor PpIX. However, despite two predominant biological processes that reduce intracellular PpIX having been identified, i.e., PpIX bioconversion and efflux, it remains to be seen whether targeting these two PpIX-reducing processes will result in any clinical benefits. Furthermore, the question regarding the biological basis of selective ALA-induced PpIX tumor disposition is yet to be addressed. Reduced tumor FECH activity is so far the most common answer to this question, although recent studies have failed to establish a correlation between tumor PpIX levels and FECH or any particular heme biosynthesis enzyme and porphyrin transporter [105,127,166]. It is likely that enhanced ALA-PpIX in tumors is a polygenic tumor phenotype determined by a group of metabolic pathways. As heme biosynthesis is deeply intertwined with glucose and iron metabolism, high PpIX tumor accumulation is certainly related to or may even be the consequence of altered glucose and iron metabolism in tumors [167]. Uncovering the interconnection between porphyrin, glucose, and iron metabolism will let us better understand abnormal porphyrin metabolism as part of global tumor-related metabolic alteration.

Finally, the success of ALA and its derivatives as tumor diagnostic and therapeutic agents comes from decades of research by biochemists, bioengineers, clinicians, photobiologists, photo-physicists, and pharmaceutical scientists. It is this multidisciplinary collaboration that culminates in the development of ALA-based drugs that are formulated in different preparations and activated by light from stable light sources to elicit biological and physical responses for therapeutic purposes. It is expected that continued collaborative research on ALA among professionals from different disciplines will bring more successes in the years to come.

## Figures and Tables

**Table 1:** ALA and its derivative drugs currently approved by the U.S. FDA.

Drug Name	Drug Formulation	Approval Time	Indications
Levulan	20% ALA hydrochloride solution	1999	Topical application for PDT of actinic keratoses
Metvixia	16.8% methyl aminolevulinate cream	2004	Topical application for PDT of actinic keratoses
Ameluz	10% ALA hydrochloride nano-emulsion gel	2016	Topical application for lesion-directed and field-directed PDT of actinic keratoses
Cysview	2 mg/mL hexaminolevulinate hydrochloride solution	2010	Intravesical instillation for the detection of non-muscle invasive papillary bladder cancer
Gleolan	ALA hydrochloride powder	2017	Oral administration for fluorescence-guided surgery of high-grade gliomas

## Data Availability

Not applicable.
